# Domestic

**Published:** 1840-11-28

**Authors:** 


### DOMESTIC.

*Division of the Muscles of the Eye for Strabismus.* By J. H. Dix, M. D., Boston.

*Division of Internal Rectus.*

*October* 16, 1840. Miss Mary M. C., æt. twenty-three, of Boston, has squinted from birth with both eyes, the left being more decidedly turned in, the edge of the cornea of which frequently reaches the inner canthus. She can on closing the right, turn the left out nearly as far as the other, but cannot keep it fixed there. Vision has always been weak, and she is conscious that this eye does not assist in seeing.

Drs. Reynolds, Jeffries, Hooper, Bethune, and Charles Ware, were present. The eyes being small and the patient very timid, I found it necessary to control the globe by fixing a double hook through the conjunctiva into the tunica albuginea about a line and a half from the edge of the cornea towards the inner canthus. By this means the eye being fairly everted, a fine, sharp hook was passed through the conjunctiva, and an incision made about half way between it and the double hook. A blunt hook was brought under the muscle, and a division made with scissors in the muscle about half an inch from the cornea, which Mr. Guthrie, whose large experience renders his authority decisive, says is preferable to a division of the tendon. On opening both eyes after the trifling hæmorrhage had ceased, the left eye is observed to be straight, the squint of the right being as before. Apply compress wet with cold water. Keep both eyes covered.

17*th.* No pain, but complains that the eye feels heavy. Bandage removed.

22*d.* There is a considerable fungous growth from the place of the incision, which is cut off with scissors.

30*th.* Miss C. has been working at her trade as a tailoress for several days past, and says that after the day's work she finds the eye somewhat turned in. In the morning it is straight. The fungous growth has re-appeared, and requires to be touched with nitras argenti.

*Nov.* 18. Since the last date Miss C. has been obliged to apply herself sedulously to sewing, and the eye is turned in somewhat, a little more perhaps than the right, with which, as was at first stated, she squints a little. Still the eye is by no means so much turned in as before the operation; she has the privilege, as she terms it, of turning it out when she pleases, and finds it serviceable to vision, until it is fatigued by long use. She proposes to have the muscle re-divided when she has an opportunity of resting the eye for a time. The fungous growth has at length disappeared, and the redness at the inner canthus has diminished.

*Division of Internal Rectus.*

*Oct.* 16. Mrs. I., æt. twenty-six, of Boston, squinted with the right eye at nine months of age, immediately after hooping-cough. The squint is very decided, a portion of the cornea being hidden at the inner canthus. Drs. Doane, Dale, and Parkman, being present, the operation was performed as in the preceding case, except that the patient possessing considerable fortitude, I dispensed with the use of the speculum and the double hook, with which in the first case the eye was turned out. She kept the eye steadily everted a little towards the outer canthus. On removing the instruments the eye was found to be perfectly straight, and capable of turning inward very little. Compress wet with cold water to be constantly kept on the eye, both being covered at the same time. Light diet.

18*th.* Mrs. I. suffering no pain, and having had none except for about eighteen hours after the operation, and then not severe, I directed the eyes to be uncovered.

20*th.* Has had some pain, about as much as immediately after the operation, which may perhaps be attributed to her getting chilled by exposure to the night air while looking from the window at a fire. Renew application of cold water. Sulph. magnesia ℥i.

21*st.* Eye comfortable. At several times since the operation she has had double vision for an hour at a time, but she has not observed it for three days past. Vision is much clearer and stronger than before the operation, and the only difference perceptible in the eyes, which are both perfectly straight, is that the right cannot be turned to the inner canthus quite so far as the left.

*Nov.* 22. Eyes perfectly straight, and motions of both parallel, the right having a farther movement inward than immediately after the operation.

*Division of Internal Rectus of both Eyes.*

*Oct.* 17, 1840. Miss Sarah H. of Boston, squinted from birth with both eyes, very badly with the left. On September 9th I divided the tendon of the internal rectus, which was followed by an amendment of the squint, which, however, after a few days, partially returned, and though now the cornea of this eye is not so far turned in as before, she at my request submits to a second division of the muscle. With the assistance of Mr. Stone, who held the lids open without using a speculum, I operated as in the former cases, omitting the double hook. Miss H. kept the eye steadily in the required direction, and upon removing the instruments and opening the other eye, this one is found to be perfectly straight. Right eye to be bandaged, and the left turned outward.

*Oct.* 22. The left eye continues to be perfectly straight, and she has control of its motions in every respect, except that she cannot turn it quite so far into the inner canthus as the right, which squints somewhat inward. The wound has healed, leaving a very slight thickening and redness of the conjunctiva.

Miss H. having expressed a wish to try the effect of the operation on the right eye, I performed, in presence of Drs. S. Keep, Dyer, Dale, and Salisbury, the operation as in the case of the other eye, except that the tendon of the muscle was divided near the globe. There was an immediate amendment of the squint, but not so complete a restoration as in the other eye. Left eye bandaged.

*Nov.* 23. Vision is much stronger than formerly, owing, I presume, chiefly to an improvement in the left eye, which, as well as the right, continues as last described; one straight, the other slightly turned in. All redness has disappeared from the left eye, and the place of the incision is observed only on close inspection.

*Division of the Internal Rectus and Superior Oblique.*

*Oct.* 23, 1840. William Augustus S., æt. eighteen, of Salem, when four years of age had an inflammation of the right eye, during which the eye was for a long time kept bandaged, and acquired a strabismus convergens. Now the eye is so far turned inward as ordinarily to conceal not only the pupil, but nearly the whole of the cornea, it being in short the most decided inversion I have ever seen. Vision with this eye is exceedingly indistinct, patient affirming, at first, that it was blind. On further investigation, it seems that he can just discern the outlines of large objects with this eye, at the distance of six feet. The loss of vision is partly to be accounted for by a slight opacity on the upper part of the cornea, but probably depends much more on the entire disuse of the eye for fourteen years. He can, by an effort of the will, turn it out nearly in front, but cannot retain it there more than a few seconds. It was therefore necessary to evert it by means of the double hook, the operation being in every respect like that in the first case. Drs. Channing, Putnam, Morrill, and Hooper, and Dr. Gustine of New Orleans, were present. Patient fainted as soon as the division of the rectus muscle was completed. On his recovery the eye was found to be considerably less turned, but still not straight, the squint being perhaps diminished one-half. With the approbation of the gentlemen present, I then proposed the division of the superior oblique muscle, to which the patient assented. This was easily accomplished, without enlarging the incision or using the double hook, the eye being now sufficiently everted by the voluntary effort of the patient to bring the incision fairly in view. Passing the blunt hook under the conjunctiva at the upper extremity of the incision, it was readily brought round the tendon of the superior oblique, bringing it fairly into view, so as to be divided with the scissors. The eye immediately inclined slightly outward. He has still the power of turning it a little inward. A compress wet with cold water on the right eye; both eyes to be kept shut.

*Oct. 27th.* Has had no inflammation beyond the limits of the incision, which seems to be occupied by one large, smooth granulation, not sufficiently prominent to give him uneasiness or to require any application. He often speaks of the increased ability of seeing objects on his right by the aid of this eye, the vision of which he thinks is improving. He is, however, obliged to cover it when exposed to a strong light, an intolerance which must be owing to the continued absence of it, and will gradually abate. The eye is straight, and the axes of the two parallel, except when he is looking far to the right, when the right eye inclines a little to the outer canthus. He returns home, with direction to close the left eye occasionally, and exercise the right, especially in the rotation of it inward; a motion which he can perform to a much greater extent than could be expected after the division of these two muscles.

*Nov. 18th.* I have not seen Mr. S. since Oct. 27th; but now learn, from his father, that the eye is perfectly straight, and more tolerant of light, though still red at the inner corner.

*Division of Internal Rectus.*

*Nov. 5th.* Miss Margaret M., æt. twenty-four, of Boston, seventeen years ago had measles, followed by disease of the eyes, after which it was observed that both eyes squinted, the right very badly. Her mother thinks, that in consequence of close application as a pupil, and afterwards as a teacher in school, the squint has been gradually growing worse. Now the right eye is turned so far in, that the edge of the cornea is usually hidden at the inner canthus, though it can be brought out at pleasure. Vision from this eye is not so good as from the left.

Drs. Perry, Wiley, Bartlett, Bethune, and Dorr, were present. The lid was raised without a speculum, and the eye sufficiently everted by the patient to render the double hook unnecessary. The conjunctiva and cellular tissue, raised by the small sharp hook, were divided by one incision, with a small and slightly curved knife. At the suggestion of Dr. Bethune the lid was now suffered to close for a minute, the hook of course being removed from the conjunctiva. Upon raising the lid, the muscle was reached with the blunt hook as easily as in the preceding cases, and divided with the scissors. The eye became immediately straight. She has the left eye now bandaged, and is directed to turn the right eye frequently out, no application whatever being made to it.

This operation occupied not more than three minutes, including the time that the lids were closed, and was, I believe, less painful than usual, in consequence of removing the sharp hook and closing the eye before the muscle was taken up on the blunt hook. The curved knife is preferable to the straight one, inasmuch as a larger incision may be made with one cut, and when the lid is raised without a speculum, the curved knife is brought out at the upper part of the incision without interfering with the finger of the person who raises the lid.

*6th.* A slight tendency outward in the right eye, which may yet be turned in by an effort of the patient. Very little pain, but a heavy sensation in the eye. To-day the eye operated on to be turned in as far and as often as possible.

*7th.* Eye straight, and to be left to itself uncovered. Double vision yesterday, and occasionally to-day.

*17th.* A slight inclination inward. Left eye to be bandaged, and the right turned forcibly out.

*24th.* The eye is now again straight, and has been so since last date. The wound in the conjunctiva is nearly cicatrized.

When speaking of an eye as perfectly straight, it is not meant that looking in some one direction, as, for instance, far to the right or left, a trifling want of correspondence in the axes of the eyes may not be detected by a close observer; but that, looking as the person ordinarily does, at objects in front of him, the cornea is midway between the inner and outer canthus, looking forwards. With this understanding, the results of the above six cases may be thus stated. In four the squint is removed, in two it is essentially improved. In one of the successful cases a second division was made; and in one of the two partially successful cases the operation is to be repeated. The speculum was employed in three cases, and in three the lids were raised by the finger of an assistant. In two cases* the double hook was used to evert the globe, and in four it was dispensed with.

* In one of these cases, it may be remembered, it was used only to evert the globe previous to the division of the internal rectus, the superior oblique being divided without it, after the division of the first muscle had enabled the patient to turn the eye partially out.

